# Supplementary material for: MDM2 Influences ACE2 Stability and SARS-CoV-2 Uptake
Source: Viruses. 2023 Aug 18;15(8):1763. doi: 10.3390/v15081763 (PMC10459000; doi:10.3390/v15081763)
Supplement: Supplementary file 1 [file viruses-15-01763-s001.zip › Supplementary Figures.pdf]

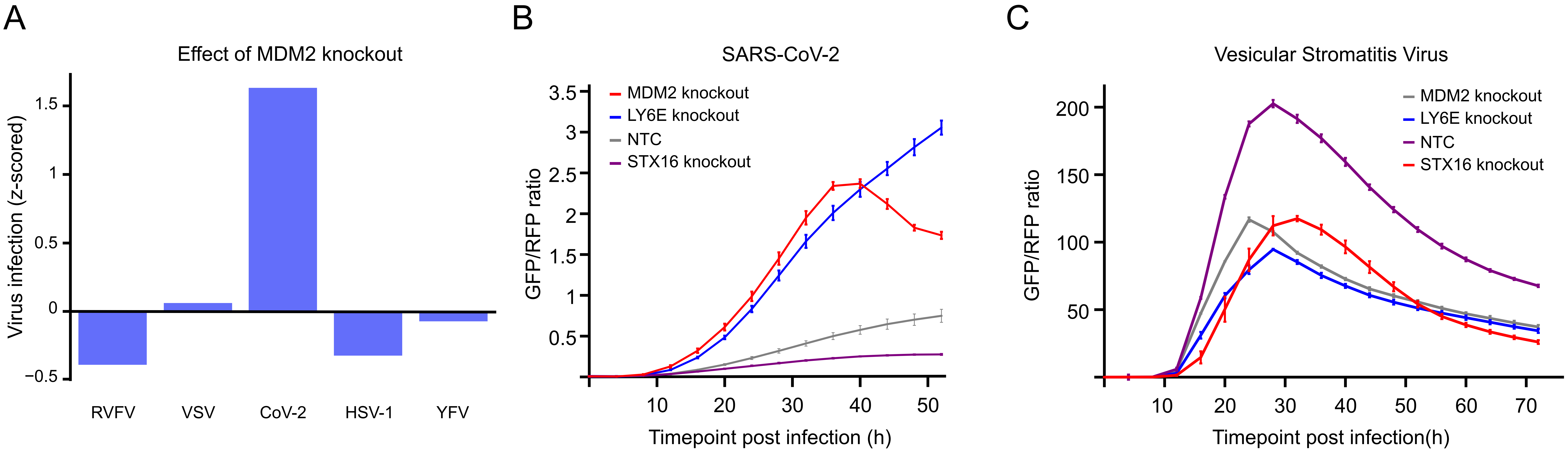

Supplementary Figure S1. Comparison of MDM2 knockout phenotype. (**A**) Related to Figure 1 - graph shows the z-score of the mean of the maximal infection value (integrated intensity GFP/mRFP ratio) for SARS-CoV-2, VSV, YFV, HSV-1, and RVFV of MDM2 knockout cells. (**B**-**C**) The indicated knockout cells or controls (NTC) were infected with SARS-CoV-2-GFP or VSV-GFP reporter viruses, and infection kinetics were measured. The virus signal (GFP integrated intensity) was normalized to the cell signal (mRFP integrated intensity) (mean +/- SD, n = 3).

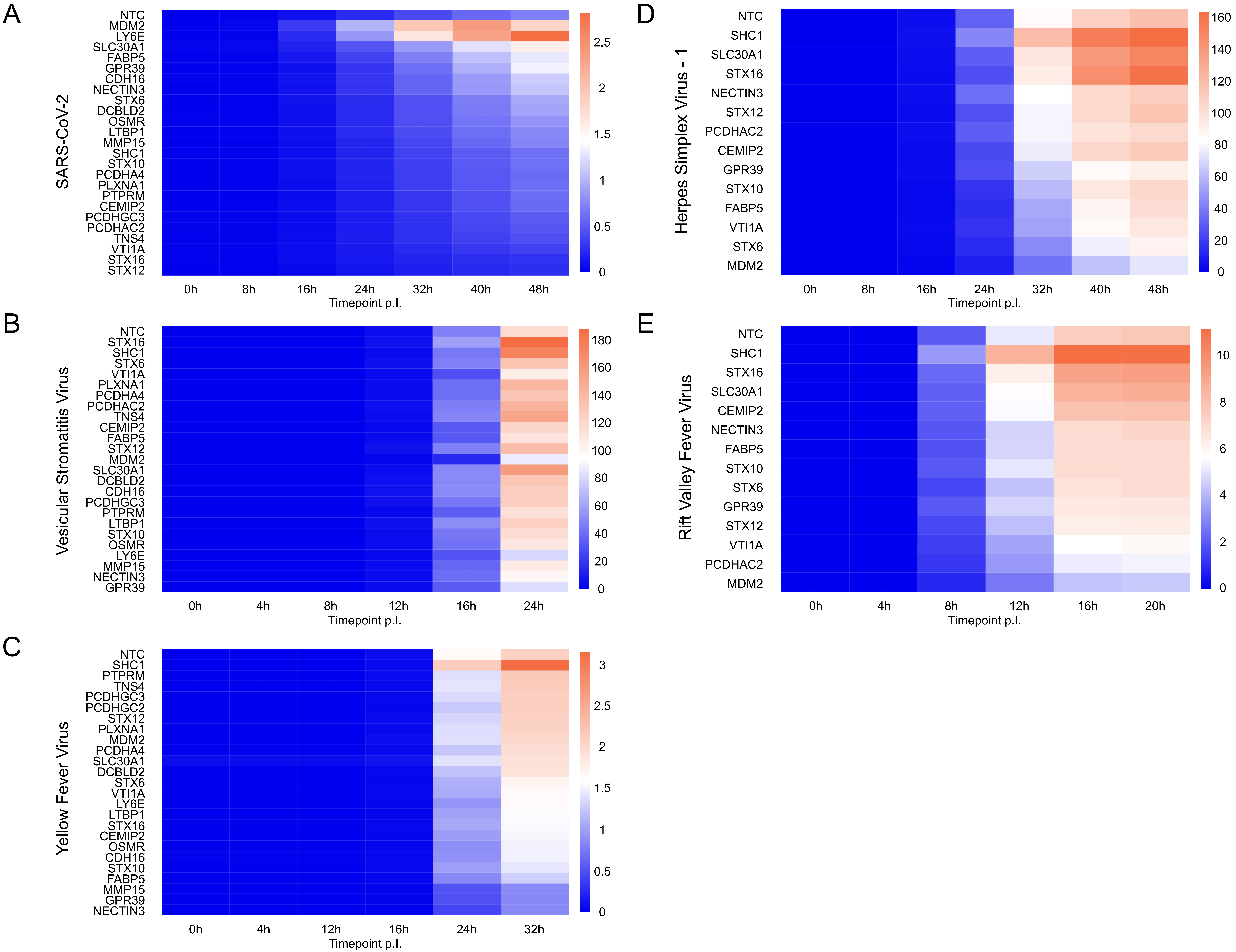
A
SARS-CoV-2
NTC
MDM2
LY6E
SLC30A1
FABP5
GPR39
CDH16
NECTIN3
STX6
DCBLD2
OSMR
LTBP1
MMP15
SHC1
STX10
PCDHA4
PLXNA1
PTPRM
CEMIP2
PCDHGC3
PCDHAC2
TNS4
VTI1A
STX16
STX12
0h 8h 16h 24h 32h 40h 48h
Timepoint p.I.
2.5 2 1.5 1 0.5 0
D
Herpes Simplex Virus - 1
NTC
SHC1
SLC30A1
STX16
NECTIN3
STX12
PCDHAC2
CEMIP2
GPR39
STX10
FABP5
VTI1A
STX6
MDM2
0h 8h 16h 24h 32h 40h 48h
Timepoint p.I.
160 140 120 100 80 60 40 20 0
B
Vesicular Stromatitis Virus
NTC
STX16
SHC1
STX6
VTI1A
PLXNA1
PCDHA4
PCDHAC2
TNS4
CEMIP2
FABP5
STX12
MDM2
SLC30A1
DCBLD2
CDH16
PCDHGC3
PTPRM
LTBP1
STX10
OSMR
LY6E
MMP15
NECTIN3
GPR39
0h 4h 8h 12h 16h 24h
Timepoint p.I.
180 160 140 120 100 80 60 40 20 0
E
Rift Valley Fever Virus
NTC
SHC1
STX16
SLC30A1
CEMIP2
NECTIN3
FABP5
STX10
STX6
GPR39
STX12
VTI1A
PCDHAC2
MDM2
0h 4h 8h 12h 16h 20h
Timepoint p.I.
10 8 6 4 2 0
C
Yellow Fever Virus
NTC
SHC1
PTPRM
TNS4
PCDHGC3
PCDHGC2
STX12
PLXNA1
MDM2
PCDHA4
SLC30A1
DCBLD2
STX6
VTI1A
LY6E
LTBP1
STX16
CEMIP2
OSMR
CDH16
STX10
FABP5
MMP15
GPR39
NECTIN3
0h 4h 12h 16h 24h 32h
Timepoint p.I.
3 2.5 2 1.5 1 0.5 0

Supplementary Figure S2. Comparative virus growth in candidate knockout cell lines. Related to Figure 1 - The indicated A549-H2B-mRFP-ACE2 knockout cells were infected with (**A**) SARS-CoV-2-GFP (MOI 3), (**B**) Vesicular Stomatitis Virus (MOI 0.01), (**C**) Yellow Fever Virus (MOI 0.1), (**D**) Herpes Simplex Virus 1 (MOI 0.1), (**E**) Rift Valley Fever Virus (MOI 0.1). Heatmaps show the average signal (GFP/RFP) in relation to time post-infection.

A B

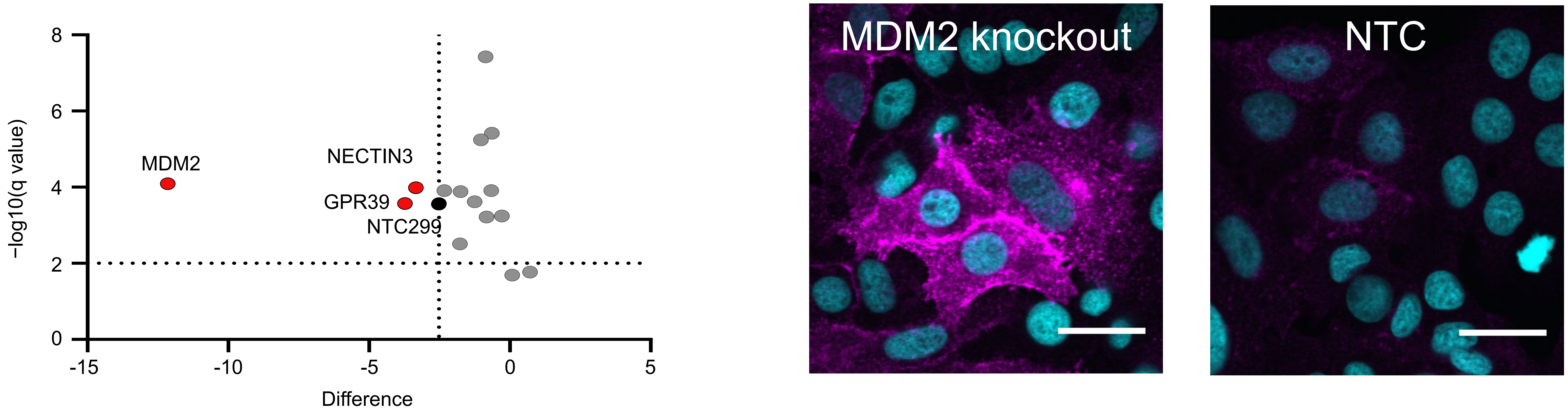

Supplementary Figure S3. MDM2 knockout increases SARS-CoV-2 RNA abundance in selected knockout cells and ACE2 protein levels (**A**) Difference in -ΔΔCt SARS-CoV-2 N RNA levels between 12 h to 24 h and corresponding q-value. The knockout cells that allowed a higher increase in transcript level as compared to the NTC are depicted in red (mean +/- SD, n = 3). (**B**) Representative confocal images of A549-H2B-mRFP-ACE2 MDM2 knockout (MDM2 KO) or control cells (NTC). ACE2 (magenta), nuclei (cyan). Scale bar equals 25 μm.

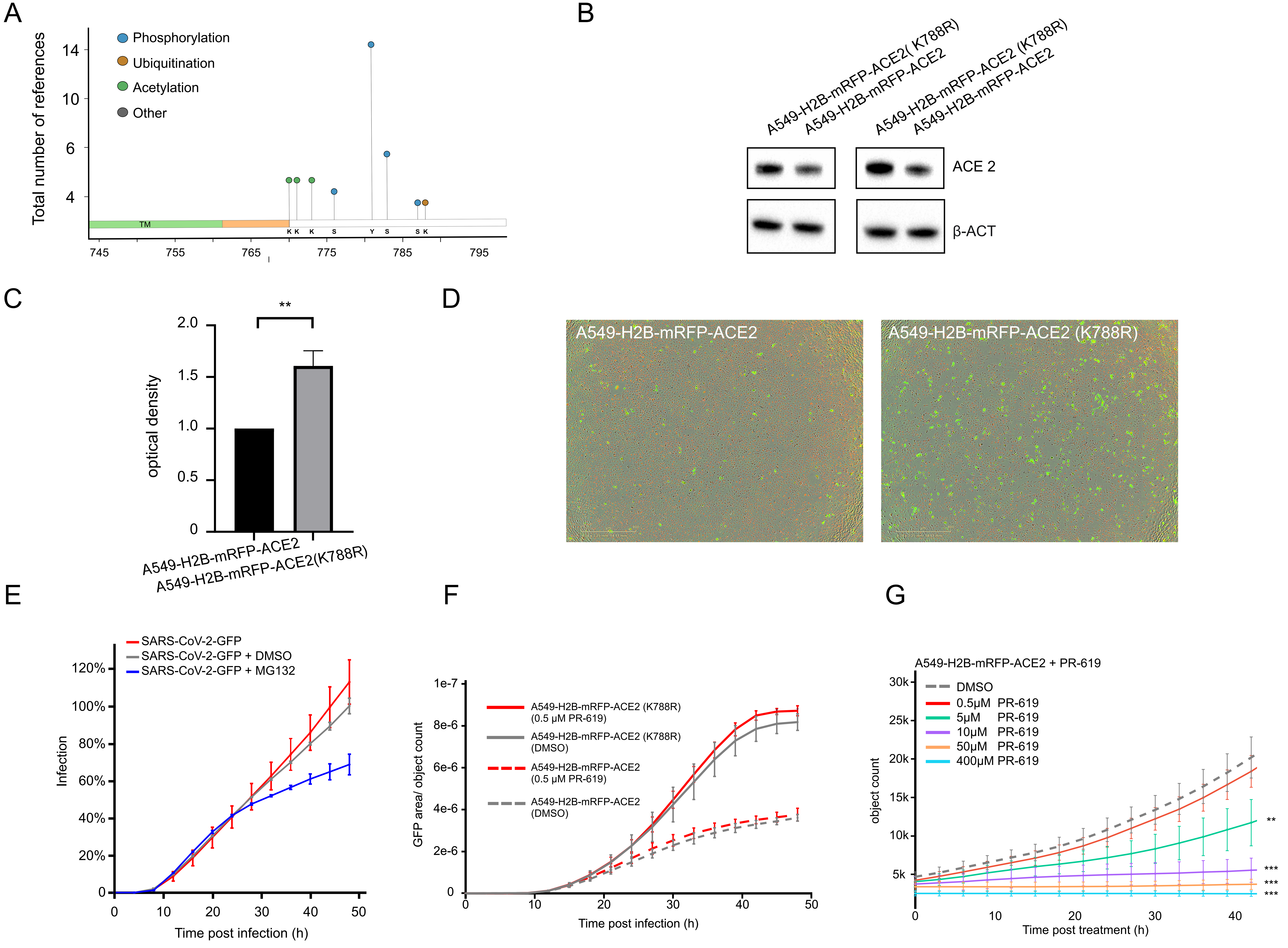

A
Total number of references
Phosphorylation
Ubiquitination
Acetylation
Other
TM
K K K S Y S S K
745 755 765 775 785 795
B
A549-H2B-mRFP-ACE2(K788R)
A549-H2B-mRFP-ACE2
A549-H2B-mRFP-ACE2 (K788R)
A549-H2B-mRFP-ACE2
ACE 2
β-ACT
C
**
optical density
A549-H2B-mRFP-ACE2
A549-H2B-mRFP-ACE2(K788R)
D
A549-H2B-mRFP-ACE2
A549-H2B-mRFP-ACE2 (K788R)
E
SARS-CoV-2-GFP
SARS-CoV-2-GFP + DMSO
SARS-CoV-2-GFP + MG132
Infection
Time post infection (h)
F
A549-H2B-mRFP-ACE2 (K788R) (0.5 μM PR-619)
A549-H2B-mRFP-ACE2 (K788R) (DMSO)
A549-H2B-mRFP-ACE2 (0.5 μM PR-619)
A549-H2B-mRFP-ACE2 (DMSO)
GFP area/ object count
Time post infection (h)
G
A549-H2B-mRFP-ACE2 + PR-619
DMSO
0.5μM PR-619
5μM PR-619
10μM PR-619
50μM PR-619
400μM PR-619
object count
Time post treatment (h)
**
***
***
***

Supplementary Figure 4. SARS-CoV-2 infection of cells expressing ACE2 or ACE2-K788R. (**A**) ACE2 structure and designated post-translational modifications of the cytosolic domain includes only one ubiquitination site (adapted from PhosPhositePlus, 2022). Lysine (K), Serine (S), Tyrosine (Y), Transmembrane (TM). (**B**) Western Blot showing ACE2 and β-actin (β-ACT) levels of A549-H2B-mRFP-ACE2 and A549-H2B-mRFP-ACE2(K788R) cells. (**C**) Western Blot quantification of ACE2 and ACE2(K788R) expressed in A549-H2B-mRFP-ACE2 and A549-H2B-mRFP-ACE2(K788R) cells shown in B and Figure 4A. ACE2 levels were normalized to the corresponding β-actin (β-ACT). The bar graph depicts the ACE2(K788R) levels (relative optical density) normalized to the ACE2. The relative optical density was determined with ImageJ, and the calculation of significance was performed with a two-sample t-test with $p < 0.01$ (**) (n = 3, +/- SD). (**D**) Epifluorescence images of A549-H2B-mRFP-ACE2 and A549-H2B-mRFP-ACE2(K788R) (24 h post-infection) cells infected with SARS-CoV-2-GFP (MOI 3). The images were acquired by a live-cell imaging platform. (**E**) MG132 inhibits SARS-CoV-2 infection. A549-H2B-mRFP-ACE2 cells were pre-treated with MG132 (1µM, 4h), DMSO control or no treatment and infected with SARS-CoV-2-GFP reporter virus (MOI:3). Virus replication was measured for 48 h post infection in a live-cell imaging platform (Incucyte S3) and analyzed by normalizing the virus signal (GFP area) to confluency (phase area). The signal of DMSO control was used to show the effect of the drug on infection (%). (**F**) A549-H2B-mRFP-ACE2 and A549-H2B-mRFP-ACE2(K788R) cells were pretreated for 6 h with 0.5µM PR-619. The cells were infected with SARS-CoV-2-GFP (MOI:3) and virus replication was measured over 48h. Virus signal (GFP) was normalized to cell number (RFP). (**G**) Cell viability screening of PR-619. A549-H2B-mRFP-ACE2 cells were pretreated 6h with PR-619 at the indicated concentrations. The cell number (RFP counts) was measured in a live-cell imaging platform (Incucyte S3). For statistical analysis a t-test between DMSO control (dashed line) and compound of interest (solid line) was performed at 24h and 48h post infection with $p < 0.01$ (**), 0.001 (***).

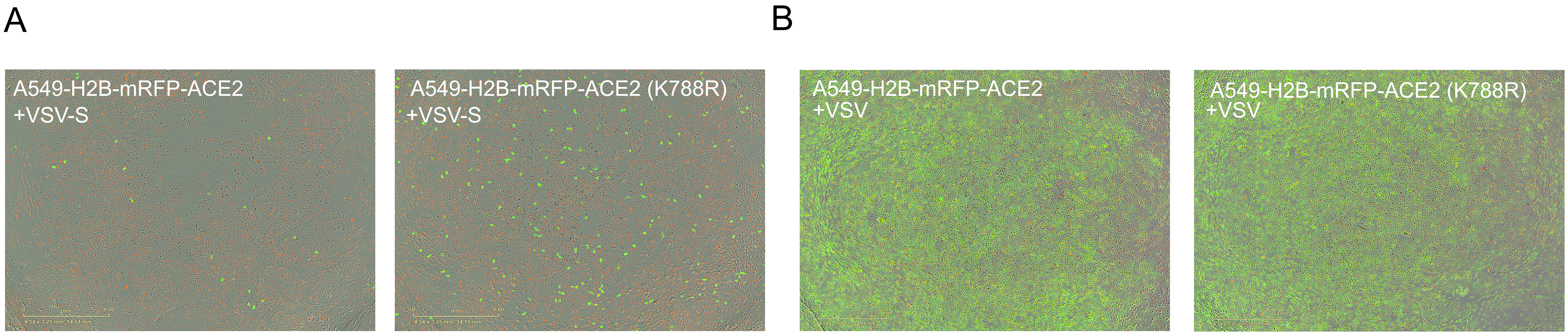

Supplementary Figure S5. ACE2-K788R increases VSV-S but not VSV infection. A549-H2B-mRFP-ACE2 and A549-H2B-mRFP-ACE2(K788R) cells were infected with either (**A**) VSV-S (MOI 3) or (**B**) VSV (MOI 0.01). Representative epifluorescence images at 12 h post-infection are shown. The images were acquired by a live-cell imaging platform.
